# Glutathione S-transferase θ1 polymorphism contributes to lung cancer susceptibility: A meta-analysis of 26 case-control studies

**DOI:** 10.3892/ol.2015.2948

**Published:** 2015-02-10

**Authors:** YAN ZHAO, BINGWEI WANG, KAI HU, JUAN WANG, SU LU, YANXI ZHANG, WEIQUAN LU, ERJIANG ZHAO, LING YUAN

**Affiliations:** 1 Department of Epidemiology and Biostatistics, College of Public Health, Zhengzhou University, Zhengzhou, Henan 450001, P.R. China; 2 Department of Radiotherapy, Affiliated Tumor Hospital of Zhengzhou University, Zhengzhou, Henan 450003, P.R. China; 3 Department of Social Medicine and Health Service Management, College of Public Health, Zhengzhou University, Zhengzhou, Henan 450001, P.R. China; 4 Henan Academy of Medical Sciences, Zhengzhou, Henan 450003, P.R. China; 5 Tianjin Medical University Cancer Institute and Hospital, Tianjin 300060, P.R. China; 6 Department of Radiotherapy, Affiliated Tumor Hospital of Zhengzhou University, Zhengzhou, Henan 450003, P.R. China

**Keywords:** meta-analysis, glutathione S-transferase θ1, polymorphism, lung cancer

## Abstract

The *GSTT1* gene encodes a key enzyme involved in the metabolism of xenobiotics and its polymorphisms have been associated with individual susceptibility to various malignancies. Numerous molecular epidemiological studies have been performed to investigate the association between *GSTT1* gene polymorphisms and lung cancer susceptibility; however, the results of previous studies were inconsistent. Therefore, the aim of the present study was to conduct a meta-analysis in order to derive a more precise estimation of the association in the East Asian populations. The meta-analysis included 7,415 lung cancer cases and 6,084 controls from 26 published studies in East Asia, which were selected from the PubMed and China National Knowledge Infrastructure databases, up to March 20, 2014. Using crude odds ratios (ORs) with 95% confidence intervals (CIs), a statistically significant association was identified between the *GSTT1* null genotype and lung cancer in the East Asian populations (OR=1.17; 95% CI, 1.09–1.25; P_heterogeneity_=0.003). Furthermore, subgroup analyses revealed that the lung cancer risk in smokers carrying the *GSTT1* null genotype was significantly increased compared with non-smokers (OR=1.71; 95% CI, 1.04–2.81; P_heterogeneity_=0.002). Thus, the *GSTT1* null genotype may increase the risk of lung cancer among the East Asian populations.

## Introduction

Lung cancer is the most common type of cancer and the leading cause of cancer-associated mortality in the world, becoming a major public health problem globally. Lung cancer accounts for 13% (1.6 million) of the total number of cancer cases and 18% (1.4 million) of mortalities in 2008 (1). In recent years, the incidence of lung cancer in Asia has increased rapidly; however, the etiological factors of the disease remain unclear. Lung cancer is known to be a complex disease; however, tobacco smoking, family history, diet and susceptible gene mutations appear to be involved in its development (1,2).

Glutathione S-transferases (GSTs) are phase II enzymes that are key in the detoxification of numerous carcinogens (3). GSTs mediate the conjugation of electrophilic compounds to glutathione, resulting in detoxification of specific environmental carcinogens and pesticides and the inactivation of polycyclic aromatic hydrocarbons, facilitating their excretion from the body (4,5). GSTs are classified into at least four genetically distinct groups: α, μ, π and θ. Theoretically, individuals lacking a specific GST enzyme may be at a particularly high risk of developing cancer, if exposed to certain genotoxicants (4,6). Among the potential mutations, the most widely known are the deletions of the *GST θ1* (*GSTT1*) or *GST μ1* (*GSTM1*) genes (null variants), which result in no enzymatic functional activity (7,8). Deficiency in *GSTT1* isoenzyme activity may predispose individuals to the effects of electrophilic carcinogens. Previous studies have proposed that *GSTT1* deficiencies may be associated with an increased susceptibility to lung cancer (9–12); however, other studies contradict this proposal (13–15). Therefore, numerous molecular epidemiological studies have been performed to investigate the potential association between the *GSTT1* gene polymorphism and lung cancer susceptibility, particularly in East Asian populations (15–17); however, the conclusions are inconsistent and occasionally contradictory. A previous study has indicated that the frequency of the *GSTT1* null genotype is higher in Asia compared with other populations (18). Thus, the present study performed a meta-analysis, including 26 eligible case-control or prospective studies (15–17,19–41), aiming to investigate the effect of the *GSTT1* polymorphism on the risk of lung cancer in the East Asian populations.

## Materials and methods

### Search strategy

Eligible studies were identified by performing a literature search on the PubMed and China National Knowledge Infrastructure (CNKI) databases (from inception to March 20, 2014) using the following keywords: ‘Glutathione S-transferase T1’, ‘GSTT1’, ‘polymorphism’, ‘lung cancer’ and the combined phrases. In addition, the references of all the relevant articles and reviews were searched for additional eligible studies. Furthermore, the full text of each potentially relevant paper was scrutinized to ensure that the following inclusion criteria were met: i) The studies had an observational (case-control or prospective) study design; ii) the authors offered sufficient data for estimating odds ratios (ORs) and their 95% confidence intervals (CIs); and, iii) the patients and controls of all the studies were East Asian (including Chinese, Japanese, South Korean, Mongolian and North Korean). In cases where multiple studies reporting on the same population data met the inclusion criteria, the study with the largest sample size was selected for subsequent analysis.

### Data extraction

In order to minimize bias, the following data were extracted from all the eligible studies by two researchers independently: First author’s surname, year of publication, country, source of the controls (population- or hospital-based studies), histological type of cancer (adenocarcinoma, squamous cell carcinoma or small-cell carcinoma), smoking status and the different genotypes in the cases and controls. In accordance with the definition used in the majority of previous studies, carriers with at least one *GSTT1* allele were defined as the ‘present’ genotype group, whereas individuals carrying no *GSTT1* alleles were classified as the ‘null’ genotype group.

### Quality assessment

The quality of the studies included in the present analysis were assessed using an adapted 10-point Newcastle-Ottawa assessment scale (NOS) (42). The quality of each study was assessed by two independent reviewers based on three broad factors: Selection (maximum score, 4), comparability (maximum score, 2) and exposure (maximum score, 4). Thus, a total quality score ranging between 0 (lowest score) and 10 (highest score) was obtained by adding all the scores. A total score of seven or greater indicated that the study was of high-quality.

### Statistical analysis

The association between lung cancer risk and the *GSTT1* polymorphism was estimated for each study using crude ORs with 95% CIs. The pooled ORs were evaluated for null vs. present genotypes and a χ^2^-based Q-statistical test was performed to assess the heterogeneity between the studies (43). P<0.05 was considered to indicate a statistically significant heterogeneity. In the case of significant heterogeneity, a random-effect model, as described by DerSimonian and Laird (44), was used to calculate pooled estimates. Otherwise, a fixed-effect model was used, as described by the Mantel-Haenszel method (45). These two models provided similar results when heterogeneity between the studies was absent. The potential publication bias was evaluated using the funnel plot and the linear regression asymmetry test, as previously described by Egger *et al* (46). P<0.05 was considered to indicate a statistical significant difference. All the statistical analyses were performed using the Statistical Analysis System (version 9.1.3; SAS Institute, Cary, NC, USA) and Review Manager software (version 5.2; The Cochrane Collaboration, Oxford, UK), with two-sided P-values.

## Results

### Eligible studies

**Figure 1:**
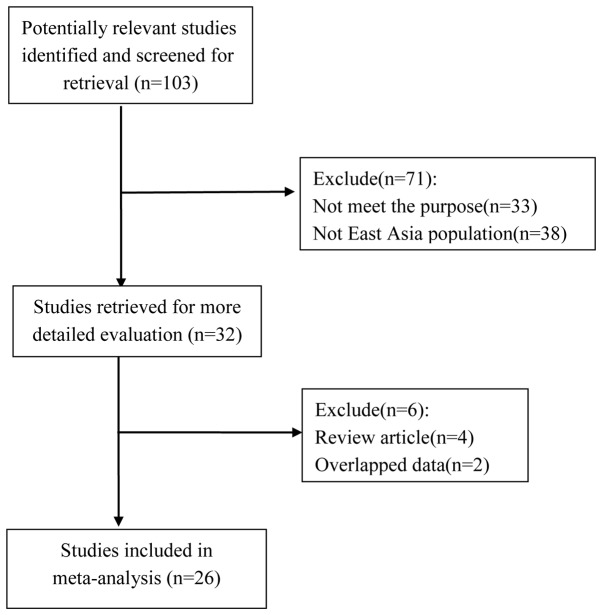
Flow chart of the selection of publications included in the current meta-analysis.

Fig. 1 indicates the process of study selection and exclusion. A total of 103 abstracts were identified in PubMed and CNKI using the aforementioned key words. Following careful review of the titles and abstracts, 32 relevant studies describing the association between *GSTT1* polymorphisms and lung cancer in the East Asian populations were selected. However, after obtaining and reading the full articles, four studies were excluded since they were review articles and two studies were excluded since they presented no data of interest or only raw data. Thus, a total of 26 eligible studies, including 7,415 lung cancer cases and 6,084 controls, were selected for inclusion in the present meta-analysis. The major characteristics of these studies are presented in Table I. The sample size range was 107–5,632 samples, while 10 studies used hospital-based control sources, 16 studies were hospital-based and two studies did not specify. In addition, sub-analyses by histological type (adenocarcinoma, 7; squamous cell carcinoma, 7; small-cell carcinoma, 3) and smoking state (smokers, 8; non-smokers, 8) were conducted. The methodological quality of the studies included is presented in Table I and the Newcastle-Ottawa assessment scale score range was 4–9, with a mean score of 6.7.

### Meta-analysis

**Figure 2:**
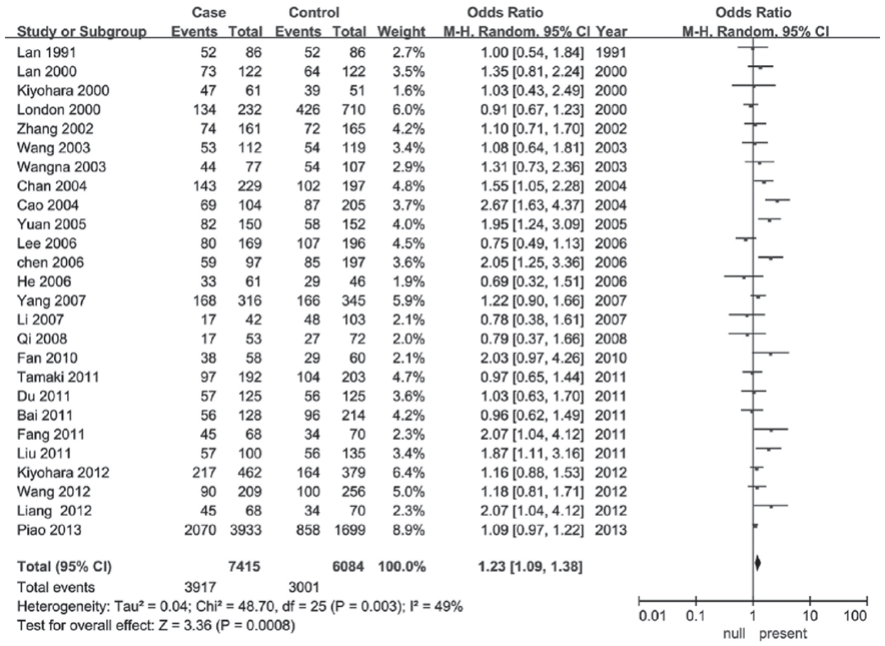
Forest plot for the association between the glutathione S-transferase θ1 polymorphism and lung cancer risk in East Asian populations.

As indicated in Table II, the pooled ORs were performed for the *GSTT1* null vs. present genotype individuals. The results indicated that individuals with the *GSTT1* null genotype were significantly associated with an increased risk of developing lung cancer compared with those carrying the *GSTT1* present genotype in the East Asian populations (OR, 1.23; 95% CI, 1.09–1.38; P_heterogeneity_=0.003; Fig. 2). When stratified by smoking status, a statistically increased lung cancer risk was identified in smokers (OR, 1.71; 95% CI=1.04–2.81; P_heterogeneity_=0.002). However, no statistically significant association was identified in non-smokers (OR=1.13; 95% CI, 0.87–1.46; P_heterogeneity_=0.42). Furthermore, in subgroup analysis by histological type, no statistically significant association was identified for all the stratified analyses. The major results of the meta-analysis and the heterogeneity test are listed in Table II.

### Publication bias

**Figure 3:**
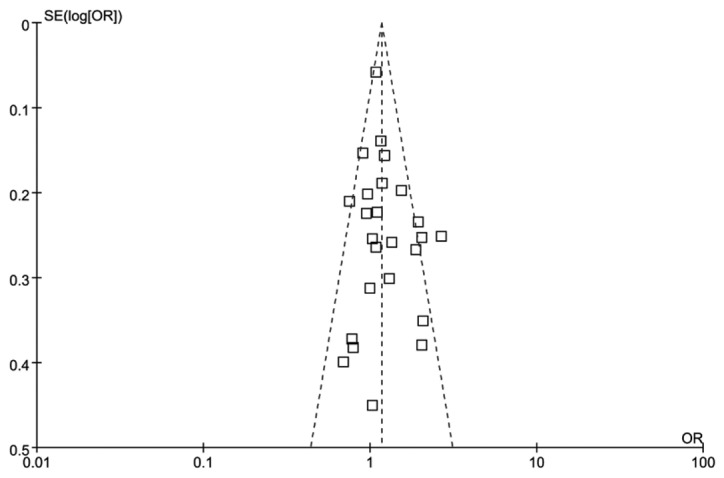
Funnel plot analysis to detect publication bias, indicating the odds ratios for the major effect of glutathione S-transferase θ1.

As demonstrated in Fig. 3, the shapes of the funnel plots appeared symmetrical in the overall populations, indicating the absence of publication bias. Furthermore, the results of the Egger’s test provided statistical evidence for the funnel plot asymmetry (t=−1.57; P=0.1265).

## Discussion

The results of the current meta-analysis indicated that *GSTT1* polymorphism was associated with an increased risk of developing lung cancer in the East Asian populations. Furthermore, in subgroups of smoking status, increased lung cancer susceptibility was identified in the smoking population.

The *GSTT1* gene is located on chromosome 22q11.2 (47). Individuals carrying homozygous deletions in the *GSTT1* genes may present an impaired ability of metabolically eliminating carcinogenic compounds and may, therefore, be at an increased risk of developing lung cancer (6). Since Deakin *et al* (48) first investigated the *GSTT1* polymorphism in lung cancer, a number of studies have been performed to evaluate the association between this homozygous deletion and the risk of developing lung cancer (4,49,50). Certain studies have indicated that this polymorphism in the *GSTT1* gene is a risk factor for lung cancer; however, this is inconsistent with the findings of other studies (51–53). Additionally, a previous study demonstrated that individuals harboring the null deletion of the *GSTT1* gene had a 2.4-fold higher risk of developing lung cancer (54). Furthermore, in a previous meta-analysis focused on the Asian population, the results were predominantly positive compared with other ethnic groups (55). The disparity in lung cancer susceptibility among different ethnicities with the *GSTT1* null genotype is consistent with a number of previous studies indicating that the frequency of the *GSTT1* deletion varies among different populations (56,57). In particular, the prevalence of the *GSTT1* null genotype is lower among Caucasians (10–20%) compared with Asians (50–60%) (58). Thus, ethnicity may be an important factor influencing the *GSTT1* gene sensitivity to lung cancer. To the best of our knowledge, the current study is the first to investigate and determine that the *GSTT1* null genotype is a risk factor of lung cancer in the East Asian populations.

Tobacco smoking is known to be one of the major risk factors of lung cancer (56). Tobacco contains a variety of carcinogens, including polycyclic aromatic hydrocarbons, N-nitrosamines and aromatic heterocyclic amines, that are transported through metabolic pathways by the GSTT1 protein (59). A number of studies have identified a higher risk of developing lung cancer in smokers carrying the *GSTT1* null genotype (52,60,61). Similarly, the present study identified that the interaction between the *GSTT1* gene and tobacco consumption has an effect on the development lung cancer. Furthermore, it has been reported that not all populations were equally susceptible to tobacco-associated carcinogens (62). The frequency of the *GSTT1* null genotype was lower in Caucasians compared with Asians, strengthening the hypothesis that polymorphisms in enzymes that metabolize tobacco carcinogens may have a strong association with ethnicity (63).

A number of previous studies have evaluated the effects of gene-gene and gene-environment interactions in lung cancer development. Chen *et al* (20) indicated that individuals carrying the *GSTT1* null genotype combined with other GST mutants may have an enhanced risk of developing lung cancer compared with individuals carrying any mutant alone. For instance, specific studies indicated that a daily diet of fruit and vegetables containing isothiocyanates may reduce the risk of lung cancer among carriers of at least one functional *GSTT1* allele (26,64,65). Brennan *et al* (65) demonstrated that the protective effect of isothiocyanate-containing vegetables was most apparent in individuals with low values of circulating GST enzymes due to the presence of null *GSTT1* alleles. In addition, the results are in accordance with various smaller studies of lung cancer, breast cancer and colorectal adenomas that demonstrated a protective effect of isothiocyanates in *GSTT1* null carriers (65). More comprehensive studies should be conducted in the future to fully understand the association between cancer risk and isothiocyanate consumption in *GSTT1* null individuals.

In the current meta-analysis, various limitations should be acknowledged. Lung cancer is known to be a consequence of multiple risk factors. For instance, lifestyle, diet, age, gender, environment and ethnicity may all contribute as the possible risk factors (48). However, due to the limited participant data provided by the individual studies, the present meta-analysis was not able to conduct a more precise assessment by ruling out those confounding factors. In addition, misclassification of cigarette consumption may have occurred due to the vague definition of cigarette consumption in certain studies. Furthermore, only published studies were included in the current meta-analysis, which may have biased the results.

In conclusion, the present study indicated that the *GSTT1* null genotype may contribute towards the increased lung cancer risk in the East Asian populations. Future large and well-designed epidemiological studies considering the potential interactions are required to confirm the results of the present meta-analysis.

## Figures and Tables

**Table I:** Characteristics of studies included in the present meta-analysis.

				Genotypic distribution							
								
				Case		Control		Newcastle-Ottawa scale			
						
Reference	Year	Country	Controls	Null	Present	Null	Present	Selection^a^	Comparability^b^	Assessment^a^	Total
Piao *et al* (17)	2013	Korean	HB	2070	1863	858	841	3	2	2	7
Wang *et al* (27)	2012	China	PB	90	119	100	156	3	2	2	7
Liang *et al* (41)	2012	China	HB	45	23	34	36	2	0	2	4
Kiyohara *et al* (15)	2012	Japan	PB	217	245	164	215	3	2	4	9
Liu (34)	2011	China	HB	57	43	56	79	3	0	3	6
Tamaki *et al* (26)	2011	Japan	PB	97	95	104	99	3	1	3	7
Fang (33)	2011	China	HB	45	23	34	36	3	0	2	5
Du (31)	2011	China	HB	57	68	56	69	3	1	2	6
Bai (29)	2011	China	HB	56	72	96	118	3	1	2	6
Fan *et al* (39)	2010	China	HB	38	20	29	31	3	2	2	7
Qi *et al* (37)	2008	China	HB	17	36	27	45	3	2	2	7
Yang *et al* (25)	2007	Korea	HB	168	148	166	179	3	2	2	7
Li (30)	2007	China	HB	17	25	48	55	3	1	2	6
Lee *et al* (23)	2006	Korea	HB	80	89	107	89	2	2	2	6
He and Tang (35)	2006	China	PB	33	28	29	17	4	0	3	7
Chen *et al* (20)	2006	China	PB	59	38	85	112	3	0	2	5
Yuan *et al* (28)	2005	China	HB	82	68	58	94	3	1	2	6
Cao *et al* (40)	2004	China	HB	69	35	87	118	2	1	2	5
Chan-Yeung *et al* (19)	2004	China	PB	143	86	102	95	4	2	2	8
Wang *et al* (5)	2003	China	NR	53	59	54	65	2	1	3	6
Wang (32)	2003	China	HB	44	33	54	53	3	0	3	6
Zhang (38)	2002	China	PB	74	87	72	93	3	2	3	8
London *et al* (24)	2000	China	PB	134	98	426	284	4	2	3	9
Lan *et al* (22)	2000	China	PB	73	49	64	58	3	2	3	8
Kiyohara *et al* (22)	2000	Japan	NR	47	14	39	12	3	2	4	9
Lan *et al* (37)	1991	China	PB	52	34	52	34	4	2	2	8

a Maximum score, 4;

b maximum score, 2.

HB, hospital-based; PB, population-based.

**Table II:** Main results of pooled ORs in the meta-analysis.

	Null versus present			
	
	Studies, n	OR	95% CI	P-value^a^
Total	26	1.23	1.09–1.38	0.003
Histological type				
SCLC	3	1.17	0.69–1.98	0.97
SCC	7	1.33	0.83–2.15	0.001
AC	7	1.24	0.98–1.57	0.10
Smoking status				
Smoker	8	1.71	1.04–2.81	0.002
Non-smoker	8	1.13	0.87–1.46	0.42

a P-value for heterogeneity.

OR, odds ratio; CI, confidence interval; SCLC, small cell lung cancer; SCC, squamous cell carcinoma; AC, adenocarcinoma.
